# Peelable Microneedle Patches Deliver Fibroblast Growth Factors to Repair Skin Photoaging Damage

**DOI:** 10.7150/ntno.79187

**Published:** 2023-06-19

**Authors:** Guojun Yang, Shiqi Hu, Haiyue Jiang, Ke Cheng

**Affiliations:** 1 Plastic Surgery Hospital, Chinese Academy of Medical Sciences & Peking Union of Medical College, 33 Ba-Da-Chu Rd., Beijing, 100144, P.R. China.; 2 Department of Molecular Biomedical Sciences and Comparative Medicine Institute, North Carolina State University, Raleigh, North Carolina 27607, United States.; 3 Joint Department of Biomedical Engineering, University of North Carolina at Chapel Hill, Chapel Hill, North Carolina 27599, United States, and North Carolina State University, Raleigh, North Carolina 27606, United States.

**Keywords:** microneedles, transdermal delivery, hyaluronic acid, photoaging, UV, fibroblast growth factors

## Abstract

**Rationale:** UV light deeply penetrates the dermis, leading to inflammation and cell death with prolonged exposure. This is a major contributor to skin photoaging. In the pharmaceutical field, fibroblast growth factors (FGFs) have gained popularity for enhancing skin quality as they facilitate tissue remodeling and re-epithelization. Nonetheless, their effectiveness is significantly hindered by limited absorption.

**Methods:** We have successfully created a dissolving microneedle (MN) patch that contains hyaluronic acid (HA) loaded with FGF-2 and FGF-21. This patch aims to improve the therapeutic efficiency of these growth factors while providing a simple administration method. We determined the performance of this patch in an animal model of skin photoaging.

**Results:** The FGF-2/FGF-21-loaded MN (FGF-2/FGF-21 MN) patch demonstrated a consistent structure and suitable mechanical properties, allowing for easy insertion and penetration into mouse skin. Within 10 minutes of application, the patch released approximately 38.50 ± 13.38% of the loaded drug. Notably, the FGF-2/FGF-21 MNs exhibited significant improvements in UV-induced acute skin inflammation and reduced mouse skin wrinkles within a span of two weeks. Furthermore, the positive effects continued to enhance over a four-week treatment period.

**Conclusion:** The proposed HA-based peelable MN patch provides an efficient approach for transdermal drug delivery, providing a promising method for improved therapeutic outcomes.

## Introduction

The skin serves as a crucial barrier against the harmful ultraviolet (UV) rays emitted by the sun. These rays are divided into three types based on their wavelengths: UVA (315-410 nm), UVB (280-315 nm), and UVC (100-280 nm) 1, 2. The atmospheric ozone layer effectively shields the Earth from UVC and a portion of UVB 3. Compared with UVA, UVB is far more energetic and harmful, as it penetrates the epidermis and superficial dermis, resulting in a series of crucial cellular events such as DNA damage, oxidative stress, and inflammation 4. Skin exposed to long-term UVB is characterized by fine and coarse wrinkles, telangiectasias, pigmentation, and rough textures, which is known as photoaging 5, 6. Furthermore, UVB irradiation is reported to cause increased levels of matrix metalloproteinases (MMPs) and decreased expression of collagen type I and type III, leading to an overall decrease in dermal collagens and an increased formation of wrinkles 7.

More effective therapies against UVB-induced skin photoaging are under investigation. Numerous formulations, such as vitamins, antioxidants, dermal fillings, and peptides have been used to protect skin from photoaging 8. In recent decades, significant advancements have been made in identifying therapeutic targets for treating photodamage, leading to the development of effective treatment regimens. Currently, there are several therapeutic options available; however, many patients discontinue their usage due to the undesirable side effects they may experience. For instance, topical retinoids, commonly used as a first-line therapy, can lead to irritation, redness, scaling, dryness, burning, stinging, peeling, and other adverse reactions 9. Chemical peeling drugs carry the risk of hypopigmentation or hyperpigmentation, skin infection, and scarring 10. Although photodynamic photo rejuvenation therapy (PDT) causes reversal of skin photoaging, patients often endure pain during the illumination process 11. The fibroblast growth factor (FGF) family encompasses proteins that are structurally similar to heparin-binding proteins and play diverse roles, especially in wound healing and angiogenesis 12. FGF-2 belongs to the FGF-1 subfamily and has the potential to stimulate granulation tissue formation, re-epithelialization, and tissue remodeling in healing-impaired wounds 13. Moreover, it has been reported to mitigate the side effects of X-irradiation 14. FGF-21, on the other hand, is a newly discovered member of the FGF-19 subfamily, and it has shown promise in lowering blood glucose levels 15. Recent studies have also revealed its anti-inflammatory and antioxidant properties 16,17. Unfortunately, both FGF-2 and FGF-21 have a short plasma half-life 17,18. Besides, it's necessary to maintain a sufficient concentration of the growth factors to allow a sustained release locally. Administration of growth factors via invasive routes using hypodermic needles enhances permeation, but this method requires professional skills and is poorly tolerated by patients due to the pain associated with injection 19. Topical treatment with FGFs only reaches the epidermis and is limited in efficacy by poor penetration through the stratum 20.

**Figure 1:**
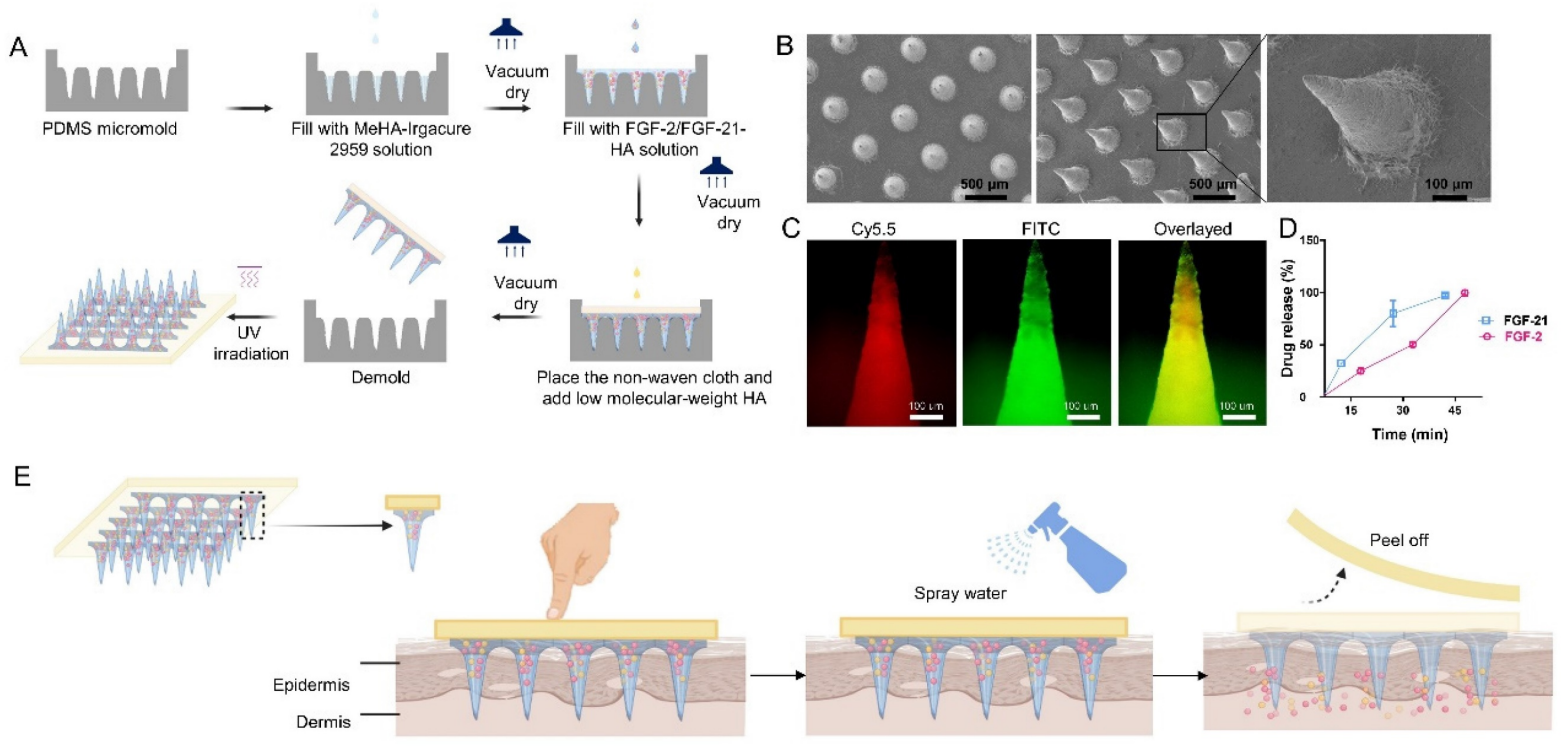
** Fabrication and characterization of the HA-based dissolving MN patch**. (A) Schematic illustration of fabrication process. (B) Scanning electron microscopy images of the MN patch. Scale bar: 500 μm. (C) Fluorescence images of a representative MN fabricated by FITC-labeled hyaluronic acid (outer layer) and PE-labeled hyaluronic acid (inner layer). Scale bar: 100 μm. (D) In vitro collective release of FGF-2 and FGF-21 from the FGF-2/FGF-21 MN patch. Data points represent mean ± SD. Error bar indicates SD. Each experimental setting has three replicates. (E) Schematic illustration of MN patch application.

In order to address the challenges associated with drug delivery, we developed a transdermal delivery system utilizing microneedles composed of hyaluronic acid (HA) for the co-delivery of FGF-2 and FGF-21, as demonstrated in **Figure 1**. As a non-sulphated glycosaminoglycan distributed abundantly in the connective tissues 21, HA possesses desirable properties in soft tissue refilling. It has been broadly used in pharmaceutical industry for its good biocompatibility, biodegradability, non-immunogenicity, and excellent water-binding ability 22. Therefore, in this study, HA was chosen as the matrix material to prepare the dissolving MN patches.

In this study, we demonstrated that in vitro, co-culture of photoaged human dermal fibroblasts (HDFs) with FGF-2 and FGF-21 led to the restoration of collagen synthesis. We characterized the drug release, skin-penetration capacity, and efficacy of the microneedles for transdermal drug delivery. Using a mouse model of UVB-induced skin photoaging, we found that both HA-based microneedle patches and FGF-2/FGF-21 loaded microneedle patches promoted collagen fiber synthesis in the dermis layer. Moreover, treatment with FGF-2/FGF-21 loaded microneedle patches further reduced skin inflammation. Our study introduces a promising approach for the efficient and sustained transdermal delivery of bioactive factors utilizing the FGF-2/FGF-21 loaded HA-based microneedle patch.

## Materials and Methods

### Materials

Sodium hyaluronate (HA) with different molecular weights were purchased from Bloomage Freda Biopharm. Human recombinant fibroblast growth factor basic (FGF-2), human recombinant fibroblast growth factor 21 (FGF-21), TGF beta-1 Human/Mouse Uncoated ELISA Kit, Alexa Fluor dyes, MTT cell viability reagent, PBS, penicillin-streptomycin and trypsin-EDTA were obtained from Thermo Fisher Scientific. Human FGF-2 ELISA kit, FGF-21 ELISA kit and Type I Procollagen ELISA was obtained from R&D Systems. Replica SILFLO and Ring Locator were brought from Clinical & Derm. All other reagents and solvents were purchased from Sigma-Aldrich.

### Cell culture and ultraviolet B irradiation of HDFs

Primary human dermal fibroblast cells (Lifeline, FC 0024) cultured in complete medium were exposed to Ultraviolet B (UVB) irradiation. The UVB dose (Philip, 311 nm, 20W/01, Germany) was 0.05 J/cm^2^ /day for 3 days.

### Cell proliferation assay

Cell Proliferation assay was performed using a MTT Assay Kit (Abcam, Cambridge, UK) in accordance with the manufacturer's protocol. In brief, HDFs were seeded in a 96-well plate at 5,000 cells per well and allowed to settle overnight, then exposed to UVB irradiation. UVB-irradiated HDFs were treated with FGF-2 and/or FGF-21 for 48 hours. Before assessment, medium was replaced by 50 µL of serum-free media and 50 µL of MTT Reagent in each well. After incubation at 37°C for 3 hours, 150 µL of MMT Solvent was added into each well and incubated on an orbital shaker for 15 minutes. Absorbance at 570 nm was read and recorded using a microplate spectrophotometer.

### Wound healing assay

HDF migration was measured in a wound healing assay, which was carried out using ibidi Culture Insert Two Wells (Ibidi, Planegg, Germany) following manufacturer's protocol. Briefly, the HDFs were seeded at 10^5^ cells per well in the inserts and allowed to settle overnight, followed by exposure to UVB (150 mJ/cm^2^). After UVB irradiation, IMEM medium containing no FGFs (control group), FGF-2 (20 ng/ml) and/or FGF-21 (10 ng/ml) were added and the cells were incubated for 24 h. The inserts were then carefully removed by sterile tweezers to start cell migration. At 0, 24, and 48 h, the scratched areas were imaged and measured using NIH ImageJ (n = 3).

### Cytochemical detection of Senescence-associated (SA) β-galactosidase (β-gal) staining

Senescence was assessed in HDFs using the Senescence Detection kit (ab65351, Abcam, Cambridge, UK) according to the manufacturer's protocol. Briefly, the cells were washed with PBS and then fixed for 15 min at RT using the fixative solution provided with the kit. Staining solution was added (1 mg/ml X-gal solution), and the cells were incubated at 37 ℃ for 18 h. After incubation, the cells were washed 2 times with PBS and the staining was imaged and measured with NIH ImageJ.

### Real-time PCR analysis

First-strand cDNA was synthesized by reverse-transcription reaction using oligo-dT primers from 1 μg of total cellular RNA (Thermo Fisher Scientific, Waltham, MA). Real-time PCR was carried out with the Power SYBR Green PCR Master Mix (Bio-Rad, Hercules, CA) using the following conditions: initial activation at 95 °C for 5 min, followed by 40 cycles of 95 °C for 15 s and 60 °C for 1 min. The primers used for real-time PCR as follows: human *β-actin*: 5'-CCCTGGCACCCAGCAC-3', 5'-GCCGATCCACACGGAGTAC-3'; human *MMP-1*: 5'-AATGTGCTACACGGATACCC-3', 5'-CTTTGTGGCCAATTCCAGGA-3', human *TNF-α*: 5'-CCCTCACACTCAGATCATCTT CT-3', 5'-GCTACGACGTGGGCTACAG-3', human *IL-1β*: 5'-AAACAGATGAAGTGCTCCTTCCAG-3', 5'-TGGAGAACACCACTTGTTGCTCCA-3'.

### Preparation and characterization of MN patches loaded with FGF-2 and FGF-21

MN patches were prepared via micromolding method 22. MeHA solution (50 mg/ml 200 kDa MeHA together with 0.5 mg/ml Irgacure 2959 in DI water) was cast into the micromold followed by vacuuming for about 1 hour. FGF-2 (0.1 μg/mL) or/and FGF-21 (0. 1 μg/mL) were mixed into MeHA solution (200 mg/mL). 100 μL solution was added onto the mold and vacuumed to fill the tips. This process was repeated three times. The dry solution outside the mold was removed gently with a blade. The estimated weight of the microneedle tips after air-drying should be 60 mg (200 mg/mL × 300 μL). Based on our studies, the tips were about 50 mg. The loss was about 15%. Since FGF-2 and FGF-21 were dissolved in MeHA solution, the loss of them was proportional to the loss of MeHA. The estimated loading content of FGF-2 and FGF-21 was 30 ng (0.1 μg/mL × 300 μL). To fabricate MN patches for the following study, a piece of non-woven fabric coated with HA solution (8 kDa, 400 mg/ml) was added to produce a robust supporting substrate, and dried overnight. Finally, MN patches were gently peeled off from the micromolds, and exposed to low-intensity ultraviolet light for 3min (360 nm, ~2 mW/cm^2^). To make MN patches for characterization, the first cast solution comprised 0.1 % (v/v) Phycoerythrin (PE, Thermofisher Scientific) in MeHA solution. The second cast comprised 0.1% FITC (Thermofisher Scientific) in HA solution (200 mg/ml 10 kDa HA).

### Characterization of MN patches

MN patches were examined using a field-emission scanning electron microscope (FESEM; JSM-6700, JEOL), and digital microscope (Leica DVM6). To assess the drug release profiles of FGF-2/FGF-21 MN patches in vitro, patches were immersed in 4 ml of DPBS at room temperature. Supernatant was collected at 15, 30 and 45 min and the concentrations of FGF-2 and FGF-21 were determined by ELISA kits according to the manufacturer's instructions (R&D Systems). The absorbance at 450 nm was measured using a microplate spectrophotometer (BioTek Instruments, Winooski, VT, USA). Separation and retention efficiency were calculated by intensity (NightOWL II LB 983 In Vivo Imaging System, BERTHOLD TECHNOLOGIES, Germany) from the dye in the MN patch before and after skin insertion and fluorescence from the dye on the rat skin.

An Instron rheometer (Instron, USA) was used to test the mechanical compression of the microneedle patch. MN patches were approached and pressed at 0.1 mm/s using a stainless-steel plate with a load capacity of 10 N 23.

### Animal study

All animal work was compliant with the Institutional Animal Care and Use Committee (IACUC) of North Carolina State University. For the topical experiment, the mouse dorsal skin (6-week-old hairless male SKH1 mice, Charles River Laboratories) was irradiated with UVB every other day for 8 weeks as previously described 24-26. The total irradiated UVB volume was approximately 80 MED. After UVB irradiation, 12 mice were randomly divided into four groups: (a) apply with MN patches; (b) apply with FGF-2/FGF-21 MN patches; (c) apply with FGF-2/FGF-21 HA solution; (d) apply with saline (control). For each mouse, the whole back skin was divided into at least three parts to be analyzed.

### Skin roughness evaluation

Skin replica was performed using a two-part mixed-type replica agent (SILFLO, Clinical & Dermal, TX, USA) according to the manufacture's instruction. A stereomicroscope was used to photograph the replica. Skin wrinkles were evaluated based on their average roughness (Ra), calculated using the roughness calculation function of ImageJ (NIH).

### Histological staining

Skin tissue were removed and fixed in 10% formalin solution, dehydrated overnight with a 20% (w/v) sucrose solution, embedded with optimum cutting temperature (OCT) compound embedding agent, and then stored at -80°C until later use for H&E staining, Masson's trichrome (MT) staining and analysis via immunofluorescence (IF) staining.

For IF staining, frozen sections were warmed at RT for 30 min and fixed in ice cold acetone for 5 min before staining. Samples were incubated with primary antibody and secondary antibodies (Alexa Fluor 488 anti-rabbit IgG, Invitrogen, CA, USA). The cells were counterstained with ProLong® Gold antifade reagent, and images were obtained using an immunofluorescence Microscope (Leica SP2, Frankfurt am Main, Germany).

The antibodies used were as follows: COL1A1 polyclonal antibody (COL1A1 antibody (diluted at 1/100; PA5-29569; Invitrogen), and elastin polyclonal antibody (diluted at 1/200; ab 21610; Abcam, Cambridge, UK).

### Cytokine array and western blot analysis

Cytokine array analysis was performed using a Mouse Apoptosis Antibody Array (AAM-APO-1) (RayBiotech, GA, USA) according to the manufacturer's instructions. Briefly, the membranes were placed in the plastic tray provided in the kit, and then 200 μL of the samples mixed was incubated overnight at 4°C. Then the membranes were washed with a wash buffer and incubated with biotinylated antibody cocktail for 2 hours at room temperature. After washing, signals were detected using a BioRad Gel Doc XR+ Documentation system (Bio-Rad, USA). Spot intensities were measured using ImageJ (NIH).

For western blot analysis, skin samples were lysed on ice for 30 min in RIPA buffer (1% NP-40, 150 mM NaCl, 10 mM Tris-HCl at pH 8.0, 1 mM EDTA) with a complete protease inhibitor (Sigma-Aldrich). Total proteins were separated by SDS-polyacrylamide gel, transferred to PVDF membranes (Millipore, Billerica, MA) and probed with primary antibodies. The antibodies used were as follows: GAPDH monoclonal antibody (diluted at 1/2,000; G8795; Sigma-Aldrich), Caspase 3 polyclonal antibody (diluted at 1/500; C8487; Sigma-Aldrich), phospho-Caspase 9 polyclonal antibody (diluted at 1/500; SAB4504126; Sigma-Aldrich), HSP 27 polyclonal antibody (diluted at 1/1,000; PA5-78010; Invitrogen), COL1A1 polyclonal antibody (diluted at 1/2,000; PA5-29569; Invitrogen), Goat anti-Mouse IgG HRP conjugated (diluted at 1/5,000; AP308P; Merck), and Goat anti-Rabbit IgG HRP conjugated (diluted at 1/5,000; AP307P; Merck). Chemiluminescence was detected using the Clarity ECL substrate kit (Bio-Rad, USA). Protein levels were quantified using Image Lab software (BioRad) and samples were normalized to GAPDH.

### Statistical Analysis

The experimental data in this study were presented as mean ± standard deviation. Comparisons among more than two groups were performed using one-way ANOVA followed by post hoc Bonferroni test. Single, double, triple, and quadruple asterisks represent p < 0.05, 0.01, 0.001, and 0.0001, respectively. All analyses were performed using GraphPad Prism 7 software (San Diego, CA, USA).

## Results and Discussion

### Fabrication and Characterization of FGF-2/FGF-21-loaded MN patch

We developed a microneedle (MN) patch loaded with FGF-2/FGF-21 using a three-step micro-molding process (**Figure 1A**). The MN patch was composed of a 10×10 array of needles, each with a height of 500 μm and a center-to center distance of approximately 500 μm (**Figure 1B**). The structure was visualized using the fluorescence image of a representative MN patch with FITC-labeled shell and PE-labeled-core (**Figure 1C**). The conic shape and sharp tips make it easy to insert into the skin.

The loading capacity is a determining factor of drug delivery efficiency. Therefore, we determined the maximum loading capacity of the FGF-2/FGF-21 MN patch by cutting the needles, weighing them, and then dissolving them in PBS. We then quantified the amount of drug released using ELISA kits. (**Figure 1D**).

The mechanical strength of the microneedles was verified through compression tests (**Figure S1**). The degradation time of the HA-based MN needles plays a critical role in determining the pattern of drug release and the frequency of application. To predict the degradation time of MN needles, we conducted *ex vivo* experiments (**Figure S2**). Upon removing the base of the MN patch, an array of spots corresponding to the needle patch was observed, indicating that the needles could be applied with minimal skin damage (**Figure 1E**, Supplemental Video 1). These results indicate that microneedles can effectively deliver drugs to the dermis.

Effective delivery of growth factors directly to the skin has been a major challenge due to their limited ability to permeate the skin barrier. This limitation has hindered the use of growth-factor-like proteins in the treatment of skin diseases. However, microneedle patches have shown promise in enhancing transdermal drug delivery in various medical fields 27. Hydrogels, with their low mechanical properties and shorter degradation duration compared to metals and polymers, are particularly well-suited for rapid drug release. The high-water content of HA hydrogels also makes them an ideal carrier for hydrophilic proteins, while their polymer network density allows for controlled drug release 28. By piercing the skin, the high-molecular-HA-based tip of the microneedle patch absorbs body fluid and releases pre-loaded FGFs to the affected area, providing a potential solution to the challenges of growth-factor delivery to the skin.

### Effects of FGF-2 and FGF-21 on the proliferation and migration of UVB-irradiated HDFs

**Figure 2:**
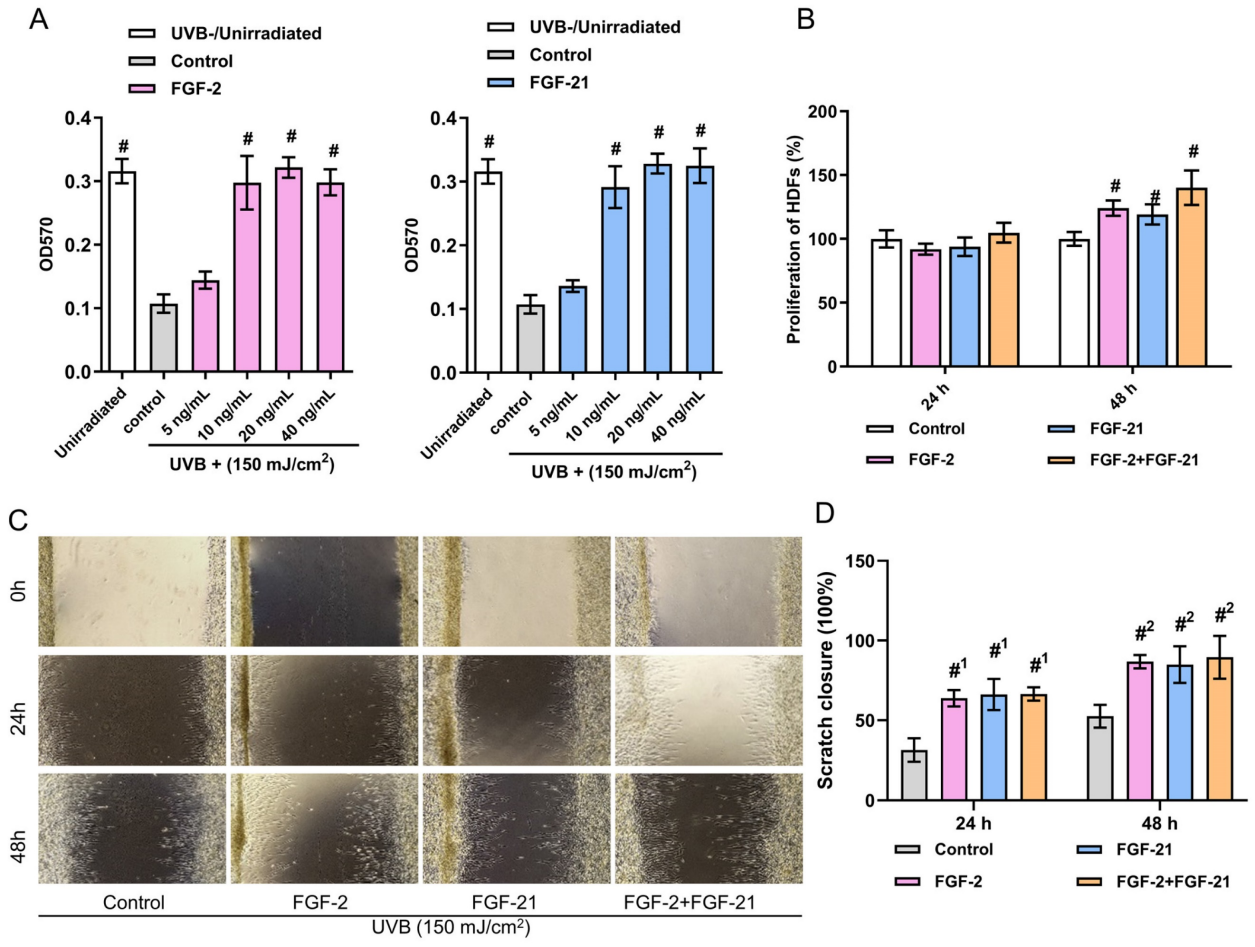
** Proliferation and immigration of HDFs.** (A) Cell viability of different concentrations of FGF-2 or FGF-21 treated HDFs. (B) HDF proliferation with the treatment of FGF-2 and/or FGF-21. (C) Wound recovery rates of UVB-exposed HDFs treated with FGF-2, FGF-21, and FGF-2/FGF-21 modeled by cell scratch assay and (D) closure rates over time. Data points represent mean ± SD. Error bar indicates SD. Each experimental setting has three replicates. # means significantly different compared with the control group (#p < 0.05); #1 means comparing to the 24 h control group and #2 means comparing to the 48 h control group.

Skin is an essential barrier that protects internal tissues from the external environment. Long-term UVB exposure can lead to a rough and fragile appearance, wrinkle formation and the adverse remodeling of the dermal extra cellular matrix (ECM) 29. Evidence suggests that reduced collagen synthesis and inhibited cell proliferate ability in HDFs are directly related to these changes 30. To simulate the UVB-induced photoaging, we irradiated commercial human dermal fibroblasts (HDFs) with 150 mJ/cm^2^ UVB. As illustrated in **Figure 2A**, cell viability of UVB-irradiated HDFs were higher with the treatment of FGF-2 (20, and 40 ng/ml groups) or FGF-21 (10, 20, and 40 ng/ml groups) for 48 hours than that of the control group (normal medium). No concentration-dependence was observed. Thus, we used 20 ng/ml FGF-2 and 10 ng/ml FGF-21 as the optimal concentrations in the present study.

Then, we investigated the effects of FGF-2 and/or FGF-21 on the proliferation, migration, and function of UVB-irradiated HDFs *in vitro*. After culturing for 48 hours, FGF-2 and/or FGF-21 notably promoted the proliferation of cells compared to the control group; besides, cells treated with a mixture FGF-2 and FGF-21 displayed a higher proliferation rate than that of FGF-21 treated group (**Figure 2B**). Assessment of migration was conducted by a wound healing assay (**Figure 2C&D**). Compared to the control group, all treated groups showed a faster rate of wound recovery. FGF-2 has garnered significant attention due to its impact on various cells involved in wound healing. Its ability to activate FGF receptors triggers signaling pathways that involve phosphoinositide 3-kinase (PI3K)/protein kinase B (Akt) and mitogen-activated protein kinase (MAPK)/extracellular signal-regulated kinase (ERK), all of which contribute to cell mitosis 31. Consequently, FGF-2 promotes the formation of granulation tissue and angiogenesis, leading to matrix synthesis and remodeling 32. FGF-21 belongs to the FGF-15/19 subfamily and exerts its biological effects through interactions with FGFR1 and βKlotho, as evidenced by *in vivo* studies. Although FGF-2 also binds to FGFR1, no evidence suggests that it competes with a receptor docking site for FGF-21 31,33.

### Effects of FGF-2 and FGF-21 on the senescence and collagen synthesis of UVB-irradiated HDFs

**Figure 3:**
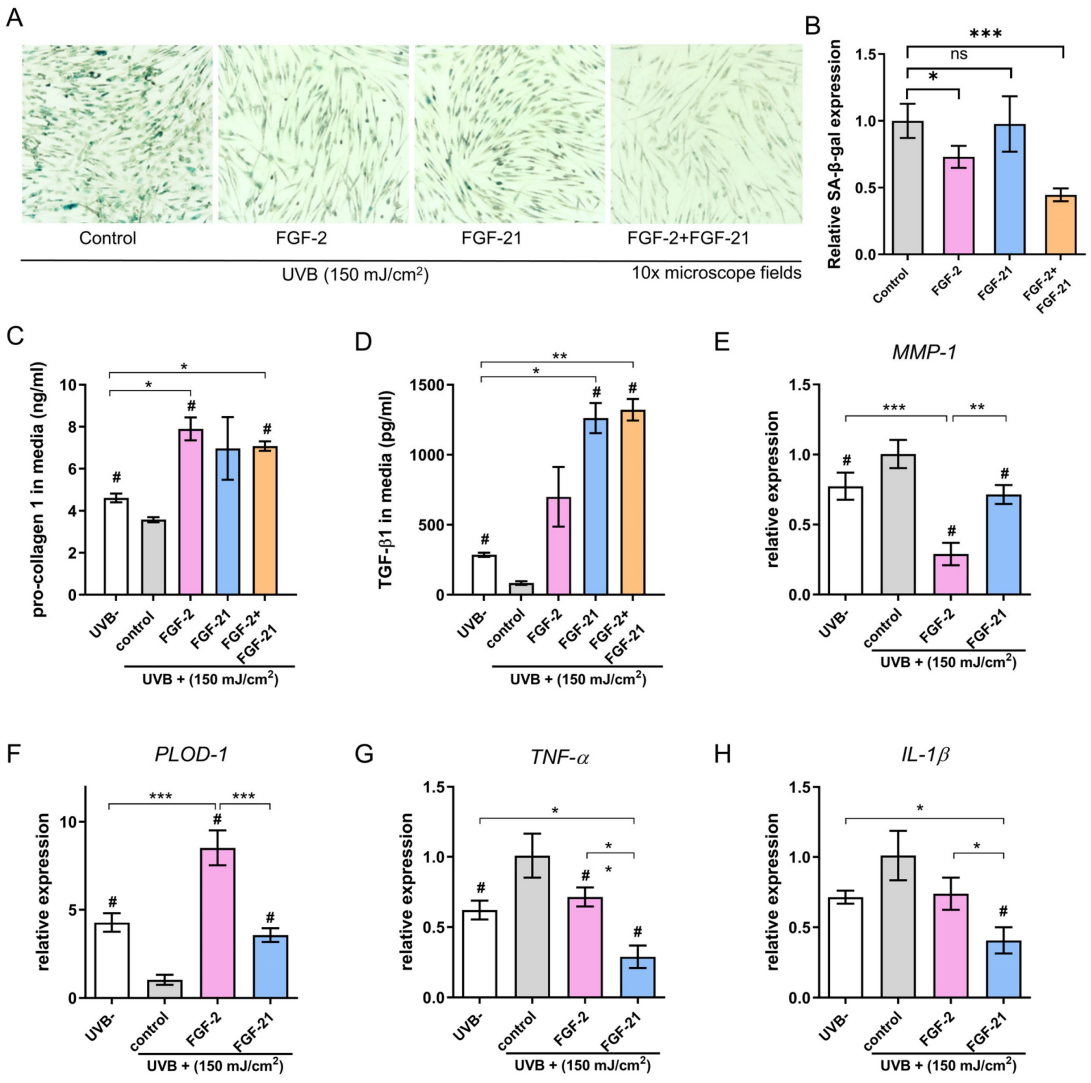
** Effects of FGF-2 and FGF-21 on HDFs.** (A) Senescence-associated (SA) β-galactosidase (β-gal) staining in UVB-exposed HDFs after treatment with FGF-2, FGF-21, and FGF-2/FGF-21 and (B) relative expression of SA-β-gal. (C-D) Secretion levels of Pro-collagen type I and TGF-β1 in HDFs with different treatments. Cell culture medium was collected and analyzed by ELISA (n=3). (E-H) Quantification of MMP-1, pro-collagen type I (PLOD-1), TNF-α, and IL-1β mRNA expression in HDFs. Data points represent mean ± SD. Error bar indicates SD. Each experimental setting has three replicates. # means significantly different with the control group (#p < 0.05); * refers to significant differences (p < 0.05) between the indicated groups.

Next, we performed SA-β-gal staining 48 hours post-UVB and treatment to evaluate the therapeutic effects of FGF-2 and/or FGF-21 on UVB-induced cell aging. As shown in Figure** 3A&B**, the number of SA-β-gal-positive cells was decreased in all treated groups compared with the control group, suggesting that both FGF-2 and FGF-21 can reverse the UVB-induced cell senescence. Furthermore, the FGF-2/FGF-21 treated group showed less SA-β-gal-positive-stained cells than that of the FGF-2 treated group, implying that FGF-21 may be a critical factor in anti-cellular senescence.

We evaluated the production of type I procollagen (pro-COL 1) and TGF-β1 using enzyme-linked immunosorbent assay (ELISA). UVB-irradiated HDFs were incubated with FGF-2 (20 ng/ml) or FGF-21 (10 ng/ml) for 48 hours before the test. UVB-irradiated HDFs treated with normal media served as a control. UVB-irradiation greatly decreased the type I procollagen concentration in the HDFs. Meanwhile, treatment with FGF-2 or FGF-2 /FGF-21 significantly up-regulated the type I procollagen production in UVB-irradiated HDFs, presenting a marked higher type I procollagen concentration, comparing with control and UVB un-irradiated groups (**Figure 3C**). As depicted in **Figure 3D**, cells treated with FGF-21 or FGF-2 /FGF-21 exhibited increased TGF-β1 secretion, while treatment with FGF-2 alone did not significantly affect TGF-β1 secretion. mRNA expression levels of collagen synthesis proteins (*PLOD-1* and *MMP-1*) and inflammatory cytokines (*TNF-α* and *IL-1β*) were quantified in four groups (**Figure 3E-H**). The results indicated a decrease in the mRNA levels of *TNF-α* and *IL-1β* in the FGF-21 treated UVB-irradiated HDFs; together with a decrease mRNA level of *MMP-1* and an increase in that of *PLOD-1* in the FGF-2 treated group as compared to the control skin.

Inflammation is a major factor in UVB-induced skin photoaging and therefore a good target for inhibiting such skin damage. The cells are known to release a variety of inflammatory cytokines during UV-induced skin damage, including tumor necrosis factor (TNF)-α and interleukin-1 (IL-1). As a multifunctional polypeptide member of the transforming growth factor beta superfamily, TGF-β1 has been described primarily as an anti-inflammatory cytokine. It is involved in diverse biological processes by regulating the production of cytokines and ECM components 34. Recent studies demonstrated that FGF-21 functions in alleviating inflammation and in the regulation of oxidative stress and autophagy-induced apoptosis 35, which is consistent with our findings. Development of skin wrinkles has been closely related to MMPs. Collagen degradation activated by MMP-1 in fibroblasts greatly compromises the skin integrity and makes it vulnerable to environment 36. Topical administration of the MMP inhibitor CGS27023 was previously proven to help reduce UVB-induced damages, including wrinkle development, gelatinase activation, damage to basement membranes, epidermal hyperplasia, and cutaneous collagen breakdown 37. In our *in vitro* model, UVB irradiation increased *MMP-1* expression together with decreased *PLOD-1* expression, and this effect was blocked by FGF-2.

Taken together, we found that FGF-21 can repress the inflammation induced by UVB irradiation, while FGF-2 can help photoaged HDFs regain the collagen synthesis ability. The proliferation capacity of UVB-photoaged was greatly promoted when the cells were treated with a combination of FGF-2 and FGF-21, with a minimum working concentration of 20 ng/ml and 10 ng/ml, respectively.

### Effects of FGF2/FGF-21-loaded MN patch on Hairless mice with UVB-induced skin photoaging

**Figure 4:**
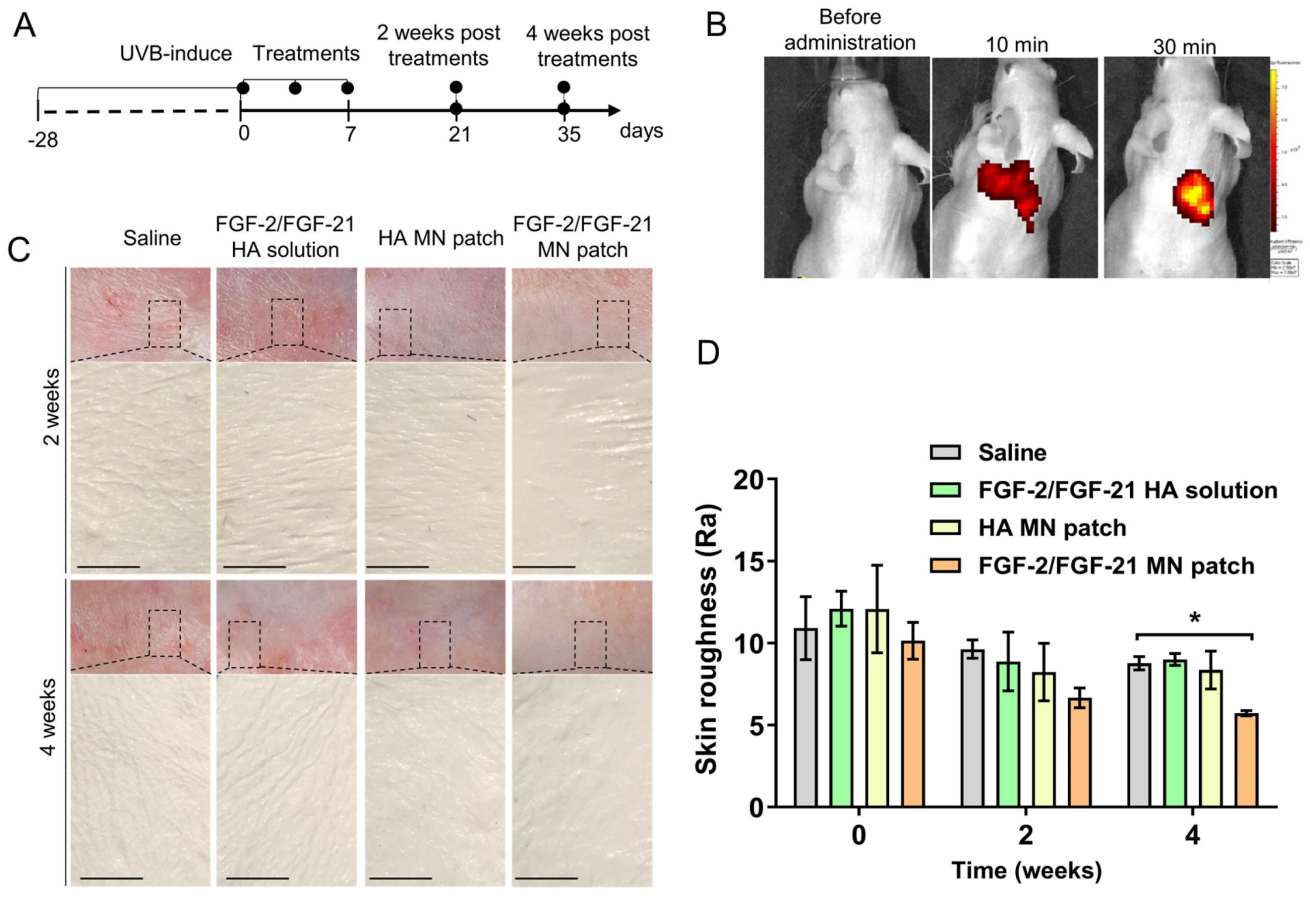
** Effects of HA MN patch, FGF-2/FGF-21 MN patch, and FGF-2/FGF-21 HA solution on skin improvement in UVB-induced hairless mice.** (A) Timeline of the mouse study. (B) In vivo release test of PE/FITC labeled-labeled MN patch. (C) Representative photographs of dorsal skin of mice and microscopic observation of replicas after 2 and 4 weeks post-of-treatment (POT). (D) Variation of skin roughness in different groups. Data points represent mean ± SD. Error bar indicates SD. Three independent biological replicates were performed. * Refers to significant differences (p < 0.05) between the indicated groups. n.s. means no significant difference.

To evaluate the *in vivo* drug release efficiency, PE/FITC-labeled HA MN patches were applied on the dorsal skin of nude mice and the base was removed (**Figure 4A-C**). The bioluminescent signal detected by an *In vivo* Imaging System (IVIS) suggested that after applied for 10 min, 38.50 ± 13.38 % of the pre-loaded PE/FITC was released, which was not significantly altered by a prolonged administration (56.11±0.60 % after 30min).

The results indicated that the HA-based MN patch could penetrate the skin and be dissolved rapidly, and the FGF-2/FGF-21 loading capacity of needles was promising to show therapeutic efficacy *in vivo*.

We next investigated the effectives of FGF2/FGF-21-loaded MN patch treatment on improving UVB-induced wrinkles in a nude mouse model. Mice were randomly divided into four groups after 8 weeks of UVB-exposure (total amount of 80 MED): HA-MN patch, FGF-2/FGF-21 MN patch, FGF-2-FGF-21 HA solution and saline. Notable differences in skin contours were observed in the MN patch treated groups stating from 2 weeks post of the treatment (POT), and we evaluated the skin condition with skin replicas, H&E staining, Masson Trichrome (MT) staining and immunofluorescence staining in the first 4 weeks POT.

Wrinkle formations in four groups were assessed with skin replicas as previously described 38. As shown in Figure 4 D, deep and wide wrinkles formed after UVB exposure. Compared to the saline group, thinner wrinkles were observed in all three treatment groups at 2- and 4-weeks POT. Efficacy of FGF-2/FGF-21 and HA in improving skin state was proved. Wrinkle measurement using the replica suggested that the Ra (average roughness) value was significantly decreased in FGF-2/FGF-21 MN patch group at 4 weeks POT, compared to saline group and FGF-2/FGF-21 HA solution group (**Figure 4D&E**).

**Figure 5:**
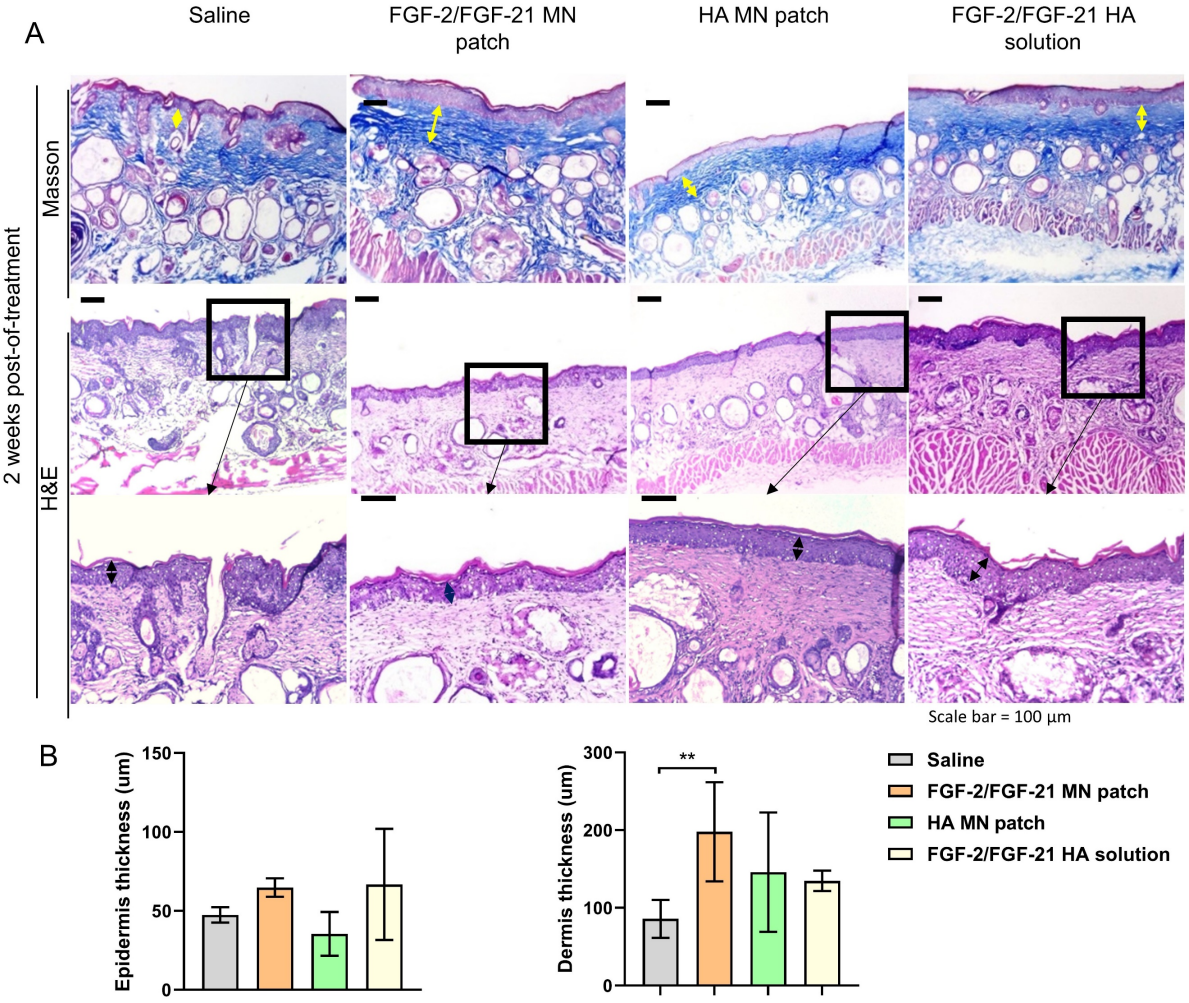
** Histological analysis of the dorsal skin of treated and untreated UVB-induced hairless mice 2 weeks post-of-treatment.** (A) Masson's Trichrome staining of 4 groups 2 weeks post-of-treatment and corresponding H&E staining. From left to right: application of HA MN patch, FGF-2/FGF-21 MN patch, FGF-2/FGF-21 HA solution and saline. Scale bar: 100 μm. (B) Epidermal and dermal thickness analysis 2 weeks post-of-treatment.

**Figure 6:**
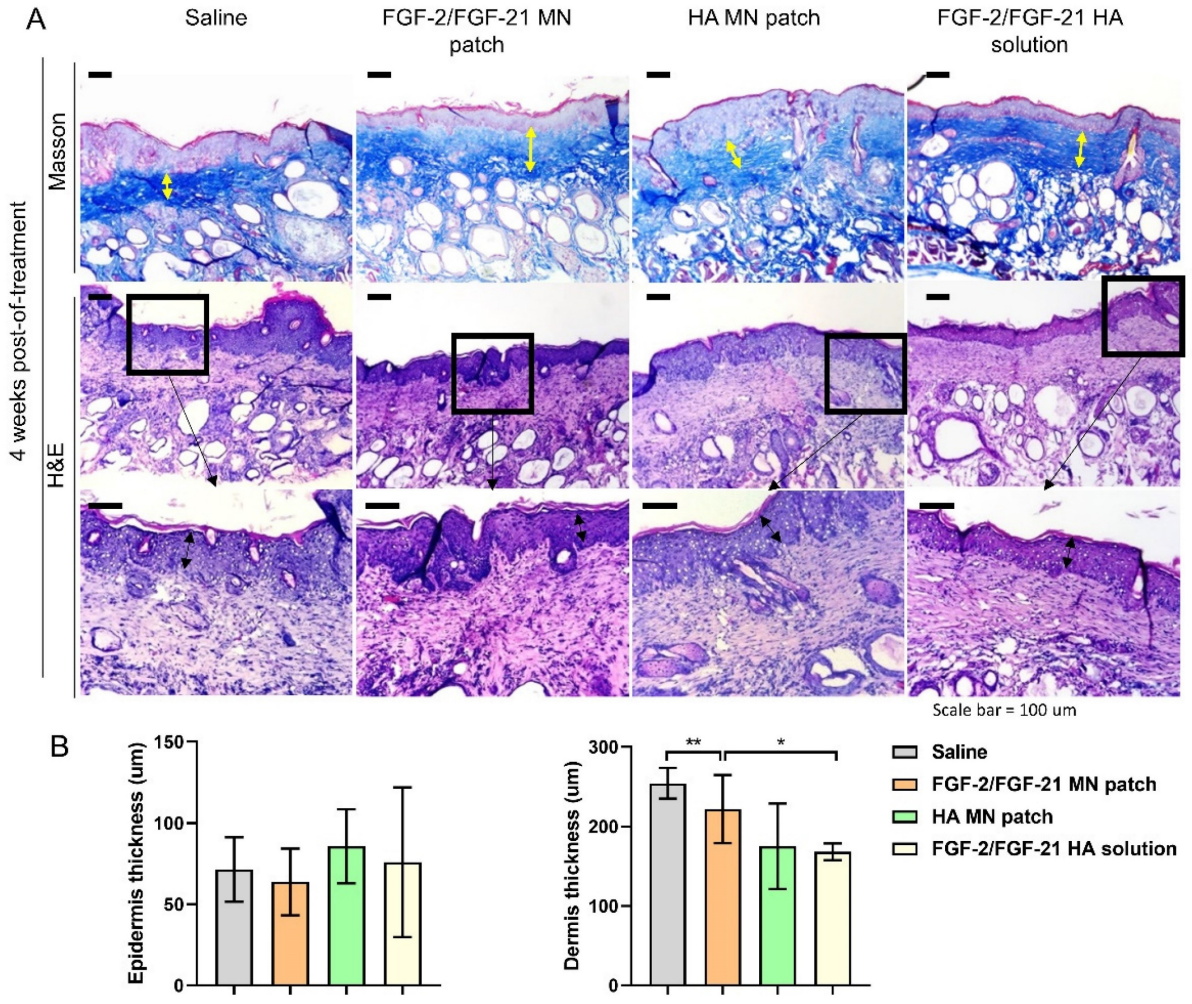
** Histological analysis of the dorsal skin of treated and untreated UVB-induced hairless mice 4 weeks post-of-treatment.** (A) Masson's Trichrome staining of 4 groups 4 weeks post-of-treatment and corresponding H&E staining. From left to right: application of HA MN patch, FGF-2/FGF-21 MN patch, FGF-2/FGF-21 HA solution and saline. Scale bar: 100 μm. (B) Epidermal and dermal thickness analysis 4 weeks post-of-treatment.

Skin remodeling was further assessed by H&E staining and Masson Trichrome (MT) staining (**Figure 5**). MT staining showed the deposition and organization of collagen fibers, suggesting an irregular, fragmented and disrupted arrangement of collagen fibers in the saline group. Thinner skin tissue with more inflammatory cells was observed in the saline group by H&E staining (**Figure 5 & 6**). Compared to saline group, FGF-2/FGF-21 MN patch group showed a significant increase in the dermal thickness due to hyperplasia of dermal fibroblasts. Additionally, increased dermal thickness was found in FGF-2/FGF-21 MN patch group, comparing with FGF-2/FGF-21 HA solution group, at 4 weeks POT. Hyaluronic acid (HA) is a major non-proteinaceous ECM component abundantly distributed in human skin. The strong hydrating properties and excellent biocompatibility make it widely used in formulations for intradermal injections to improve wrinkles. Animal studies showed a significantly improved skin condition in both HA MN patch and FGF-2/FGF-21 MN patch groups, compared with the topical application of FGF-2/FGF-21 HA solution and saline groups.

**Figure 7:**
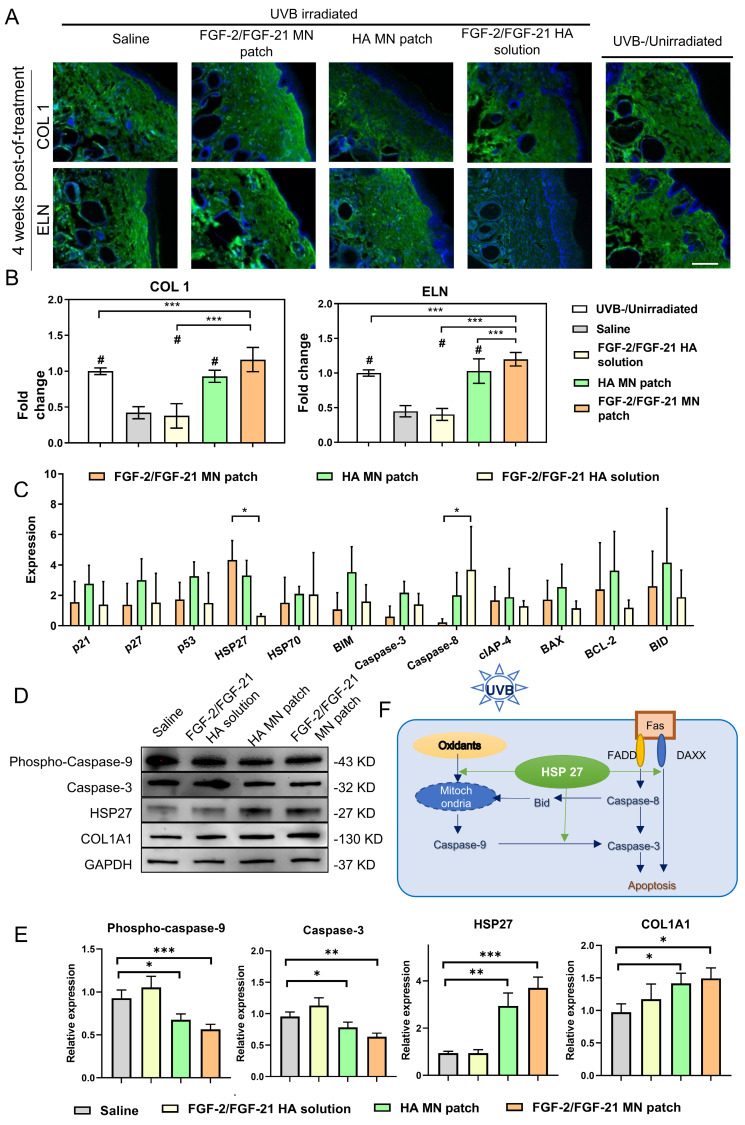
** Antiphotoaging mechanism analysis**. (A) Immunofluorescence staining of dorsal 4 weeks post-of-treatment. From left to right: application of HA MN patch, FGF-2/FGF-21 MN patch, FGF-2/FGF-21 HA solution, saline, and normal skin as a control. Scale bar: 130 μm. (B) Statistical analysis based on the IOD value and mean density captured by ImageJ software. Data points represent mean ± SD. Error bar indicates SD. At least three independent biological replicates were performed. # means significantly different with the control group (#p < 0.05); * refers to significant differences (p < 0.05) between the indicated groups. (C) Mouse apoptosis cytokine array of dorsal skin 4 weeks post-of-treatment by densitometric analysis (relative expression normalized to saline group). Differentially expressed proteins are marked by rectangles of different colors. Data points represent mean ± SD. Error bar indicates SD. Three independent biological replicates were performed. (D) Western blot of dorsal skin of different groups 4 weeks post-of-treatment and (E) quantification of Western blot results. (F) Schematic illustration of the mechanism of FGF-2/FGF-21 MN patch treatment. Error bar indicates SD. * refers to significant differences (p < 0.05) between the indicated groups.

Human skin mainly consisted of collagen and elastin fibers, which provide strength and elasticity to the tissue. As shown in **Figure 7A**, in the normal skin (sham, unirradiated), collagen and elastin are tightly bound to each other in a regular basket-weave pattern. However, in photoaged skin, the dermal elastic fiber network was destructed and replaced by thick, tangled, and tortuous fibers (saline group); and will finally result in a cluster of amorphous and dystrophic elastotic material throughout the dermis 39. Both HA MN patch treated group and FGF-2/FGF-21 MN patch treated group exhibited an increased deposition and better organization of COL 1 and ELN, compared to the other treatment groups. Medical needling has become a valuable technique in dermatology for restoring the healthy appearance of hypertrophic burn scars. The process of tissue regeneration stimulated by microneedles leads to the synthesis and deposition of collagen, ultimately improving the skin's appearance 40. Furthermore, needling has been shown to affect vascularization by promoting angiogenesis during the wound healing process 41,42. In contrast, the use of topical treatment alone failed to improve skin condition** (Figure 7B)**, indicating poor penetration of growth factors through the epidermis and the greater efficiency of dermal delivery via a microneedle patch.

### Anti-photoaging effects of FGF-2/FGF-21 MN patch treatment

To determine the molecular mechanisms of FGF-2/FGF-21 MN patches in ameliorating UVB-induced skin photoaging, we conducted cytokine array on skin samples after treatments. As shown in **Figure 7C-F**, expression levels of apoptosis proteins were analyzed between groups. FGF-2/FGF-21 MN patch group expressed a significantly higher level of HSP27, meanwhile a lower level of caspase-8. Thus, we deduce that such FGF-2/FGF-21 MN patch treatment can alleviate UVB-induced cell apoptosis by inhibiting caspase-3 expression via up-regulating HSP27. We evaluated the caspase-9, caspase-3, HSP27, and collagen type 1 expression levels in all groups to further investigate the molecular mechanism.

Cutaneous exposure to UV irradiation causes the generation of reactive oxygen species (ROS), which leads to oxidative DNA damage and consequent apoptosis 43. To minimize tissue injury, Hsp27, a member of the small heat shock protein family (sHSP), functions as an important molecular chaperone. Studies have demonstrated that Hsp27 can protect against the oxidation of proteins and interact with and inhibit caspase-3 activation, thus suppressing apoptosis 44. Recent research has reported the "switch" function of Hsp27 in UVB-induced DNA damage 45, where it enhances Akt stabilization and phosphorylation of p21, resulting in the translocation of p21 from nuclei to cytoplasm, removing cell cycle arrest, and promoting normal proliferation. Our findings are in consistent with this mechanism.

## Conclusion

The role of FGF in the skin anti-aging process has been extensively researched and validated. However, the penetration and absorption of large molecules into the dermis were very limited due to the cutaneous barrier. MN patch is a promising, minimally invasive approach to drug delivery, especially for repeat dosing 46,47.

In this study, we demonstrated the development of a low-cost, HA-based, peelable MN patch to overcome the challenges of transdermal delivery of peptides/proteins, even enabling self-administration. We have shown that the FGF-2/FGF-21 MN patch has good therapeutic effects in alleviating UVB-induced skin photoaging by reducing inflammation and promoting collagen synthesis both* in vitro* and *in vivo*. In addition to demonstrating the activation of collagen and elastin synthesis via FGF-initiated signaling pathways 36, 48, our studies have proven the synergistic effect of FGF-2 and FGF-21. Consequently, we highlighted the role of Hsp27 in the anti-aging process, suggesting it as a promising target for skin anti-aging therapy.

Collectively, we successfully developed a peelable HA microneedle patch for skin anti-aging, providing a feasible and painless alternative to syringe injection. Unlike topical treatments, the microneedle tips allow for efficient delivery of growth factors directly to the dermis, stimulating collagen production and reducing fibroblast senescence. The potential for therapeutic use in cosmetics is significant. In future studies, we plan to optimize microneedle patch geometry, material, force of application, patch wearing time, and other variables to improve delivery efficiency.

## Supplementary Materials

Supplementary video 1. Application of a microneedle patch on a hairless mouse.

Supplementary figures.
