# Supplementary material for: Peelable Microneedle Patches Deliver Fibroblast Growth Factors to Repair Skin Photoaging Damage
Source: Nanotheranostics. 2023 Jun 19;7(4):380–92. doi: 10.7150/ntno.79187 (PMC10327422; doi:10.7150/ntno.79187)
Supplement: Supplementary file 2 — Supplementary figures. [file ntnov07p0380s2.pdf]

Supplementary Materials

Peelable Microneedle Patches Deliver Fibroblast Growth Factors to Repair Skin Photoaging Damage

Guojun Yang[123], Shiqi Hu[23], Haiyue Jiang[1], Ke Cheng*[23]

[1]*Plastic Surgery Hospital, Chinese Academy of Medical Sciences & Peking Union of Medical College, 33 Ba-Da-Chu Rd., Beijing, 100144, P.R.China.*

[2]*Department of Molecular Biomedical Sciences and Comparative Medicine Institute, North Carolina State University, Raleigh, North Carolina 27607, United States.*

[3]*Joint Department of Biomedical Engineering, University of North Carolina at Chapel Hill, Chapel Hill, North Carolina 27599, United States, and North Carolina State University, Raleigh, North Carolina 27606, United States.*

*Corresponding author.

Email addresses: ke_cheng@ncsu.edu.

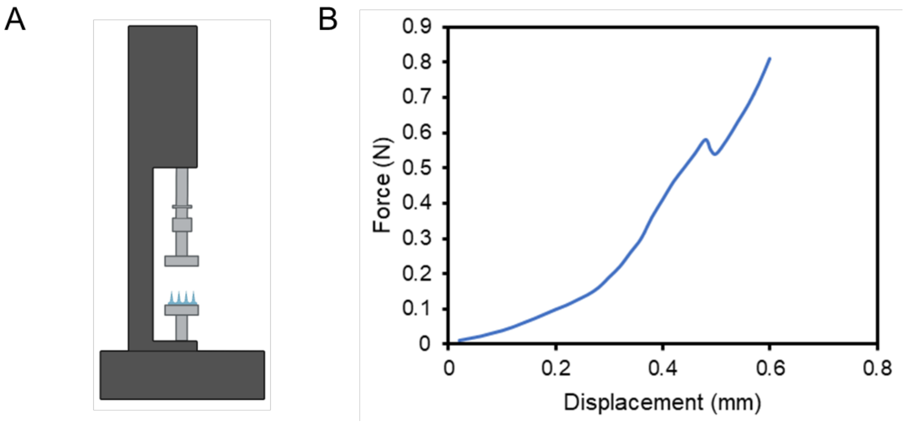

Figure S1. Mechanical strength of microneedle patch. A. Schematic illustration of the compression test for a single microneedle patch. B. Graphs of displaced distances and applied forces for the microneedle patch with a needle tip height of 500 μm.

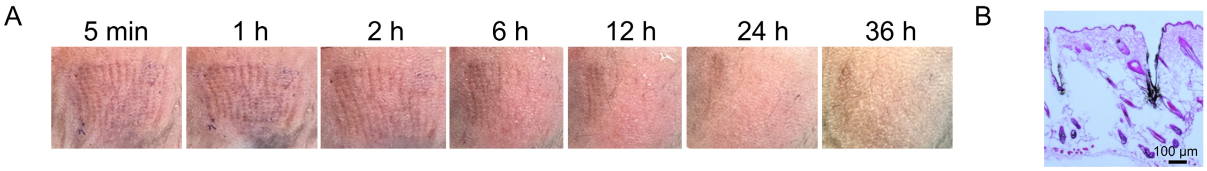

Figure S2. Images of mice skin over time frame following the application of microneedle. From left to right, 5 min, 1 h, 2 h, 6 h, 12 h, 24 h and 36 h after application. The needle tips are invisible in 36 h after application. (B) H&E staining of the skin 5 min after microneedle application.
